# 3′,4′-Dimeth­oxy­biphenyl-4-carbonitrile

**DOI:** 10.1107/S1600536812011464

**Published:** 2012-03-21

**Authors:** Xin-Min Li, Yan-Jun Hou, Peng Mei, Wen-Yi Chu, Zhi-Zhong Sun

**Affiliations:** a College of Chemistry and Materials Science, Heilongjiang University, Harbin 150080, People’s Republic of China

## Abstract

The title compound, C_15_H_13_NO_2_, was prepared through a palladium-catalysed Suzuki–Miyaura coupling reaction. The dihedral angle between the biphenyl rings is 40.96 (6)°. The meth­oxy groups are twisted slightly out of the plane of the benzene ring [C—C—C—C torsion angles = −3.61 (18) and 12.6 (2)°]. The packing of the molecules is stabilized by van der Waals inter­actions.

## Related literature

For general background to the synthesis and properties of 3′,4′-dimeth­oxy­biphenyl-4-carbonitrile, see: Suzuki (1999 ▶); Razler *et al.* (2009 ▶); Hou *et al.* (2011 ▶). For the biological activity of biphenyl derivatives, see: Kimpe *et al.* (1996 ▶).
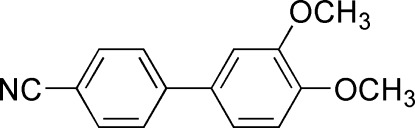


## Experimental

### 

#### Crystal data

C_15_H_13_NO_2_
*M* *_r_* = 239.26
Monoclinic, 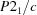

*a* = 9.1568 (10) Å
*b* = 7.7541 (8) Å
*c* = 17.6764 (19) Å
β = 96.266 (1)°
*V* = 1247.6 (2) Å^3^
*Z* = 4
Mo *K*α radiation
μ = 0.09 mm^−1^
*T* = 295 K
0.32 × 0.30 × 0.26 mm

#### Data collection

Bruker SMART APEX CCD detector diffractometer
Absorption correction: multi-scan (*SADABS*; Sheldrick, 1996 ▶) *T*_min_ = 0.973, *T*_max_ = 0.978
8149 measured reflections
2399 independent reflections
1839 reflections with *I* > 2σ(*I*)
*R*_int_ = 0.020

#### Refinement

*R*[*F* ^2^ > 2σ(*F* ^2^)] = 0.036
*wR*(*F* ^2^) = 0.104
*S* = 1.05
2399 reflections
166 parameters
H-atom parameters constrained
Δρ_max_ = 0.15 e Å^−3^
Δρ_min_ = −0.12 e Å^−3^

### 

Data collection: *APEX2* (Bruker, 2004 ▶); cell refinement: *SAINT* (Bruker, 2004 ▶); data reduction: *SAINT*; program(s) used to solve structure: *SHELXS97* (Sheldrick, 2008 ▶); program(s) used to refine structure: *SHELXL97* (Sheldrick, 2008 ▶); molecular graphics: *SHELXTL* (Sheldrick, 2008 ▶); software used to prepare material for publication: *publCIF* (Westrip, 2010 ▶).

## Supplementary Material

Crystal structure: contains datablock(s) I, global. DOI: 10.1107/S1600536812011464/bx2401sup1.cif

Supplementary material file. DOI: 10.1107/S1600536812011464/bx2401Isup2.cdx

Structure factors: contains datablock(s) I. DOI: 10.1107/S1600536812011464/bx2401Isup3.hkl

Supplementary material file. DOI: 10.1107/S1600536812011464/bx2401Isup4.cml

Additional supplementary materials:  crystallographic information; 3D view; checkCIF report

## Figures and Tables

**Fig. 1.**
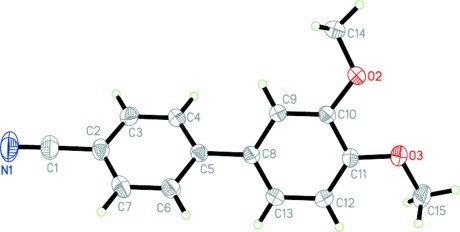
The molecular structure of the title compound I, with displacement ellipsoids drawn at the 50% probability level.

## supplementary crystallographic information

### Comment

Considerable interest shows the palladium-catalyzed Suzuki-Miyaura coupling reaction and the biological activity of biphenyl derivatives (Suzuki, 1999; Razler *et al.*, 2009; Kimpe *et al.*, 1996; Hou *et al.*, 2011). We have prepare 3',4'-dimethoxybiphenyl-4 -carbonitrile as a potential antiviral activity compound. In the title compound, Fig. 1, the dihedral angle of the biphenyl moiety is 40.96 (6)°. The methoxy groups are slightly twisted out of the plane of the benzene ring 3.65 (12) & 12.40 (13)° at C10 and C11 position respectively. The crystal structure is stabilized by van der waals interactions.

### Experimental

To a solution of 4-bromobenzonitrile (5 mmol) and 3,4-dimethoxyphenylboronic acid (6 mmol) in 20 ml water and 20 ml methanol was added Pd(OAc)_2_ (5 mmol) and K_2_CO_3_ (10 mmol). After stirring the reaction mixture for 8 h at 318 K. The aqueous phases were extracted with 100 ml ethyl acetate. The organic extracts were washed with 200 ml saturated aqueous sodium chloride, dried over sodium sulfate, filtered, and concentrated under reduced pressure. The resulting crude material was purified *via* silica gel chromatography (petroleum ether) to afford a translucent solid in a yield of 76%. Crystals suitable for single-crystal X-ray diffraction were obtained by recrystallization from methanol at room temperature in a total yield of 36%. Analysis found: C 75.3, H 5.6, N 6.0%; C_15_H_13_NO_2_ requires: C 75.3, H 5.5, N 5.9%.^ 1^H NMR (400 MHz, CDCl_3_) 7.78 (m, 2H), 7.64 (m, 2H), 7.17 (dd, *J* = 8.3, 2.1 Hz, 1H), 7.09 (d, *J* = 2.1 Hz, 1H), 6.97 (d, *J* = 8.3 Hz, 1H), 3.96 (s, 3H), 3.94 (s, 3H).

### Refinement

H atoms bound to C atoms were placed in calculated positions and treated as riding on their parent atoms. C—H distances are in the range 0.93-0.96 Å. *U*_iso_(H) values were constrained to be 1.2U_eq_(C) (aromatic H atoms) [1.5U_eq_(C) for methyl H atoms].

### Crystal data

**Table:**

C_15_H_13_NO_2_	*F*(000) = 504
*M**_r_* = 239.26	*D*_x_ = 1.274 Mg m^−^^3^
Monoclinic, *P*2_1_/*c*	Mo *K*α radiation, λ = 0.71073 Å
Hall symbol: -P 2ybc	Cell parameters from 2577 reflections
*a* = 9.1568 (10) Å	θ = 2.9–25.8°
*b* = 7.7541 (8) Å	µ = 0.09 mm^−^^1^
*c* = 17.6764 (19) Å	*T* = 295 K
β = 96.266 (1)°	Block, colorless
*V* = 1247.6 (2) Å^3^	0.32 × 0.30 × 0.26 mm
*Z* = 4	

### Data collection

**Table:**

Bruker SMART APEX CCD detector diffractometer	2399 independent reflections
Radiation source: fine-focus sealed tube	1839 reflections with *I* > 2σ(*I*)
Graphite monochromator	*R*_int_ = 0.020
phi and ω scans	θ_max_ = 26.0°, θ_min_ = 2.9°
Absorption correction: multi-scan (*SADABS*; Sheldrick, 1996)	*h* = −10→11
*T*_min_ = 0.973, *T*_max_ = 0.978	*k* = −9→8
8149 measured reflections	*l* = −21→21

### Refinement

**Table:**

Refinement on *F*^2^	Secondary atom site location: difference Fourier map
Least-squares matrix: full	Hydrogen site location: inferred from neighbouring sites
*R*[*F*^2^ > 2σ(*F*^2^)] = 0.036	H-atom parameters constrained
*wR*(*F*^2^) = 0.104	*w* = 1/[σ^2^(*F*_o_^2^) + (0.052*P*)^2^ + 0.1238*P*] where *P* = (*F*_o_^2^ + 2*F*_c_^2^)/3
*S* = 1.05	(Δ/σ)_max_ < 0.001
2399 reflections	Δρ_max_ = 0.15 e Å^−^^3^
166 parameters	Δρ_min_ = −0.12 e Å^−^^3^
0 restraints	Extinction correction: *SHELXL97* (Sheldrick, 2008), Fc^*^=kFc[1+0.001xFc^2^λ^3^/sin(2θ)]^-1/4^
Primary atom site location: structure-invariant direct methods	Extinction coefficient: 0.017 (2)

### Special details

**Table:**

Geometry. All e.s.d.'s (except the e.s.d. in the dihedral angle between two l.s. planes) are estimated using the full covariance matrix. The cell e.s.d.'s are taken into account individually in the estimation of e.s.d.'s in distances, angles and torsion angles; correlations between e.s.d.'s in cell parameters are only used when they are defined by crystal symmetry. An approximate (isotropic) treatment of cell e.s.d.'s is used for estimating e.s.d.'s involving l.s. planes.
Refinement. Refinement of *F*^2^ against ALL reflections. The weighted *R*-factor *wR* and goodness of fit *S* are based on *F*^2^, conventional *R*-factors *R* are based on *F*, with *F* set to zero for negative *F*^2^. The threshold expression of *F*^2^ > σ(*F*^2^) is used only for calculating *R*-factors(gt) *etc*. and is not relevant to the choice of reflections for refinement. *R*-factors based on *F*^2^ are statistically about twice as large as those based on *F*, and *R*- factors based on ALL data will be even larger.

### Fractional atomic coordinates and isotropic or equivalent isotropic displacement parameters (Å^2^)

**Table:**

	*x*	*y*	*z*	*U*_iso_*/*U*_eq_	
O3	0.14237 (10)	0.09170 (14)	0.04364 (6)	0.0626 (3)	
C8	0.55897 (13)	0.10197 (17)	0.16381 (6)	0.0431 (3)	
C9	0.51177 (13)	−0.03577 (17)	0.11570 (6)	0.0420 (3)	
H9	0.5753	−0.1271	0.1096	0.050*	
C5	0.70869 (13)	0.10066 (16)	0.20533 (7)	0.0432 (3)	
C10	0.37226 (13)	−0.03711 (17)	0.07734 (6)	0.0425 (3)	
O2	0.31442 (9)	−0.16798 (13)	0.03141 (5)	0.0546 (3)	
C11	0.27761 (13)	0.10261 (18)	0.08472 (7)	0.0475 (3)	
C13	0.46307 (14)	0.23659 (19)	0.17187 (8)	0.0526 (4)	
H13	0.4923	0.3277	0.2043	0.063*	
C4	0.82954 (14)	0.04428 (19)	0.17115 (8)	0.0520 (4)	
H4	0.8170	0.0075	0.1208	0.062*	
C3	0.96813 (15)	0.04188 (19)	0.21075 (9)	0.0568 (4)	
H3	1.0483	0.0055	0.1869	0.068*	
C12	0.32371 (15)	0.23763 (19)	0.13225 (8)	0.0554 (4)	
H12	0.2609	0.3300	0.1378	0.066*	
C6	0.73122 (15)	0.15651 (19)	0.28075 (7)	0.0522 (3)	
H6	0.6519	0.1970	0.3043	0.063*	
C7	0.86821 (15)	0.1529 (2)	0.32089 (8)	0.0585 (4)	
H7	0.8809	0.1900	0.3712	0.070*	
C2	0.98743 (15)	0.09404 (18)	0.28652 (8)	0.0552 (4)	
C14	0.40771 (15)	−0.30952 (19)	0.01883 (8)	0.0569 (4)	
H14A	0.4924	−0.2685	−0.0033	0.085*	
H14B	0.3552	−0.3904	−0.0152	0.085*	
H14C	0.4384	−0.3652	0.0664	0.085*	
C15	0.05399 (16)	0.2429 (2)	0.03800 (10)	0.0692 (5)	
H15A	0.0321	0.2764	0.0878	0.104*	
H15B	−0.0359	0.2199	0.0064	0.104*	
H15C	0.1061	0.3343	0.0160	0.104*	
C1	1.13159 (18)	0.0889 (2)	0.32852 (10)	0.0703 (5)	
N1	1.24412 (18)	0.0863 (2)	0.36292 (10)	0.1005 (6)	

### Atomic displacement parameters (Å^2^)

**Table:**

	*U*^11^	*U*^22^	*U*^33^	*U*^12^	*U*^13^	*U*^23^
O3	0.0425 (5)	0.0668 (7)	0.0749 (7)	0.0073 (5)	−0.0095 (4)	−0.0125 (5)
C8	0.0431 (7)	0.0452 (8)	0.0406 (6)	−0.0020 (6)	0.0025 (5)	−0.0018 (5)
C9	0.0407 (7)	0.0414 (7)	0.0437 (6)	0.0013 (5)	0.0044 (5)	−0.0018 (5)
C5	0.0457 (7)	0.0389 (7)	0.0440 (7)	−0.0037 (5)	0.0001 (5)	−0.0005 (5)
C10	0.0433 (7)	0.0435 (7)	0.0405 (6)	−0.0040 (6)	0.0042 (5)	−0.0042 (5)
O2	0.0467 (5)	0.0526 (6)	0.0624 (6)	−0.0005 (4)	−0.0030 (4)	−0.0167 (4)
C11	0.0381 (6)	0.0545 (8)	0.0489 (7)	0.0007 (6)	0.0001 (5)	−0.0031 (6)
C13	0.0518 (8)	0.0512 (9)	0.0536 (8)	0.0016 (6)	−0.0002 (6)	−0.0143 (6)
C4	0.0487 (8)	0.0546 (9)	0.0515 (7)	−0.0009 (6)	0.0000 (6)	−0.0093 (6)
C3	0.0471 (8)	0.0523 (9)	0.0700 (9)	0.0002 (6)	0.0014 (6)	−0.0052 (7)
C12	0.0479 (7)	0.0523 (9)	0.0650 (9)	0.0094 (6)	0.0022 (6)	−0.0118 (7)
C6	0.0526 (7)	0.0568 (9)	0.0466 (7)	−0.0028 (7)	0.0023 (6)	−0.0042 (6)
C7	0.0647 (9)	0.0609 (10)	0.0467 (7)	−0.0092 (7)	−0.0077 (7)	−0.0009 (6)
C2	0.0519 (8)	0.0429 (8)	0.0665 (9)	−0.0068 (6)	−0.0128 (7)	0.0069 (6)
C14	0.0606 (8)	0.0495 (9)	0.0602 (8)	0.0003 (7)	0.0044 (7)	−0.0145 (6)
C15	0.0512 (8)	0.0735 (11)	0.0792 (10)	0.0127 (8)	−0.0097 (7)	0.0050 (8)
C1	0.0652 (10)	0.0561 (10)	0.0830 (11)	−0.0044 (8)	−0.0216 (9)	0.0080 (8)
N1	0.0749 (10)	0.0913 (12)	0.1230 (14)	−0.0034 (9)	−0.0452 (10)	0.0111 (10)

### Geometric parameters (Å, º)

**Table:**

O3—C11	1.3681 (15)	C4—H4	0.9300
O3—C15	1.4217 (18)	C3—C2	1.392 (2)
C8—C13	1.3814 (18)	C3—H3	0.9300
C8—C9	1.4038 (17)	C12—H12	0.9300
C8—C5	1.4828 (16)	C6—C7	1.3724 (18)
C9—C10	1.3794 (16)	C6—H6	0.9300
C9—H9	0.9300	C7—C2	1.383 (2)
C5—C4	1.3880 (18)	C7—H7	0.9300
C5—C6	1.3954 (17)	C2—C1	1.4426 (19)
C10—O2	1.3695 (15)	C14—H14A	0.9600
C10—C11	1.4023 (18)	C14—H14B	0.9600
O2—C14	1.4231 (17)	C14—H14C	0.9600
C11—C12	1.3795 (19)	C15—H15A	0.9600
C13—C12	1.3870 (18)	C15—H15B	0.9600
C13—H13	0.9300	C15—H15C	0.9600
C4—C3	1.3809 (18)	C1—N1	1.1381 (18)
			
C11—O3—C15	117.49 (11)	C11—C12—C13	120.43 (12)
C13—C8—C9	118.70 (11)	C11—C12—H12	119.8
C13—C8—C5	121.20 (11)	C13—C12—H12	119.8
C9—C8—C5	120.10 (11)	C7—C6—C5	121.27 (13)
C10—C9—C8	120.72 (11)	C7—C6—H6	119.4
C10—C9—H9	119.6	C5—C6—H6	119.4
C8—C9—H9	119.6	C6—C7—C2	120.02 (12)
C4—C5—C6	118.15 (11)	C6—C7—H7	120.0
C4—C5—C8	121.65 (11)	C2—C7—H7	120.0
C6—C5—C8	120.20 (11)	C7—C2—C3	119.67 (12)
O2—C10—C9	125.07 (11)	C7—C2—C1	120.31 (14)
O2—C10—C11	115.12 (11)	C3—C2—C1	120.01 (14)
C9—C10—C11	119.81 (11)	O2—C14—H14A	109.5
C10—O2—C14	117.66 (10)	O2—C14—H14B	109.5
O3—C11—C12	124.69 (12)	H14A—C14—H14B	109.5
O3—C11—C10	115.83 (11)	O2—C14—H14C	109.5
C12—C11—C10	119.46 (11)	H14A—C14—H14C	109.5
C8—C13—C12	120.83 (12)	H14B—C14—H14C	109.5
C8—C13—H13	119.6	O3—C15—H15A	109.5
C12—C13—H13	119.6	O3—C15—H15B	109.5
C3—C4—C5	121.05 (12)	H15A—C15—H15B	109.5
C3—C4—H4	119.5	O3—C15—H15C	109.5
C5—C4—H4	119.5	H15A—C15—H15C	109.5
C4—C3—C2	119.80 (14)	H15B—C15—H15C	109.5
C4—C3—H3	120.1	N1—C1—C2	178.6 (2)
C2—C3—H3	120.1		
			
C13—C8—C9—C10	−0.17 (18)	C5—C8—C13—C12	179.36 (12)
C5—C8—C9—C10	179.33 (11)	C6—C5—C4—C3	0.6 (2)
C13—C8—C5—C4	−139.90 (14)	C8—C5—C4—C3	−179.25 (12)
C9—C8—C5—C4	40.61 (18)	C5—C4—C3—C2	1.0 (2)
C13—C8—C5—C6	40.26 (18)	O3—C11—C12—C13	179.70 (13)
C9—C8—C5—C6	−139.23 (13)	C10—C11—C12—C13	0.7 (2)
C8—C9—C10—O2	−177.57 (11)	C8—C13—C12—C11	0.9 (2)
C8—C9—C10—C11	1.74 (18)	C4—C5—C6—C7	−1.3 (2)
C9—C10—O2—C14	−3.61 (18)	C8—C5—C6—C7	178.53 (12)
C11—C10—O2—C14	177.05 (11)	C5—C6—C7—C2	0.4 (2)
C15—O3—C11—C12	12.6 (2)	C6—C7—C2—C3	1.2 (2)
C15—O3—C11—C10	−168.41 (13)	C6—C7—C2—C1	−179.64 (14)
O2—C10—C11—O3	−1.71 (16)	C4—C3—C2—C7	−2.0 (2)
C9—C10—C11—O3	178.92 (11)	C4—C3—C2—C1	178.93 (14)
O2—C10—C11—C12	177.36 (12)	C7—C2—C1—N1	0 (7)
C9—C10—C11—C12	−2.01 (19)	C3—C2—C1—N1	179 (100)
C9—C8—C13—C12	−1.1 (2)		
